# Confounding and collinearity in regression analysis: a cautionary tale and an alternative procedure, illustrated by studies of British voting behaviour

**DOI:** 10.1007/s11135-017-0584-6

**Published:** 2017-11-13

**Authors:** Ron Johnston, Kelvyn Jones, David Manley

**Affiliations:** School of Geographical Sciences, University of Bristol, Bristol, BS8 1SS UK

**Keywords:** Regression analysis, Confounding, Collinearity, Voting behaviour

## Abstract

Many ecological- and individual-level analyses of voting behaviour use multiple regressions with a considerable number of independent variables but few discussions of their results pay any attention to the potential impact of inter-relationships among those independent variables—do they confound the regression parameters and hence their interpretation? Three empirical examples are deployed to address that question, with results which suggest considerable problems. Inter-relationships between variables, even if not approaching high collinearity, can have a substantial impact on regression model results and how they are interpreted in the light of prior expectations. Confounded relationships could be the norm and interpretations open to doubt, unless considerable care is applied in the analyses and an extended principal components method for doing that is introduced and exemplified.

## Introduction

Quantitative analyses of voting behaviour are heavily dependent on regression modelling of data at both individual and ecological scales. The goal in most cases is to identify the influences on decisions whether to vote or abstain and, if the former, which party to support. The independent variables are selected to represent the expected influences based on theory (often relatively weak), prior investigations, and the local (time and space) context. In the interpretation of those regressions—usually either ordinary least squares or binomial/multinomial logistic—emphasis is placed on the sign, magnitude and statistical significance of the coefficients for the independent variables.

Such regression analyses can produce substantial insights but also have their disadvantages, some of which may be acknowledged in discussions of the output, but frequently their full import is not realised, with implications—often serious but unacknowledged—for the interpretation of the results. Many of these implications reflect the need for care not only in specifying models but also in assessing the results. This paper focuses on one issue only in that context—confounding associated with collinearity; without careful assessments of a regression outcome, misleading interpretations can result.

Collinearity (sometimes termed multicollinearity) is usually defined as when two or more independent variables included in the model are highly correlated so that the values of one can be accurately predicted by that of another. This has clear implications for the size, perhaps the sign, and also the standard error of the regression coefficients associated with those collinear variables, and hence for their interpretation. The result is frequently termed confounding, the situation when the relationship between two variables is distorted because of the strength of the relationships between either one or both of them and a third variable included in the analysis (see, for example, Kish 1959; Morabia 2011; VanderWheele and Shpitser 2013).^1^ Thus the relationship between age and abstention at an election may be confounded by the inclusion of income in the statistical modelling, if, for example, affluent young males are more likely than comparable older males to abstain but affluent young females are more likely to vote than affluent older females.

The epidemiological (Moon et al. 2000) and survey analysis (Rosenberg 1968) literatures have developed a useful classification of types of outcome relating to what happens to the relationship between the ‘exposure’ (the main predictor of interest) and the response when a third variable is introduced.

- *No confounding* occurs when the inclusion of a third variable does not change the empirical relationship between the outcome and the predictor;
- *Spurious inflation* involves an apparent relationship either disappearing or at least being attenuated when the third or ‘extraneous’ variable is controlled by inclusion;
- *Masking or suppression* is the case when the apparent absence of a relationship between predictor and outcome is spurious, so that the true strong relationship has been either reduced or cancelled because the suppressor variable has not been taken into account; and
- *Reverse interpretation* occurs if a distorter third variable is not controlled for, so that the correct interpretation is exactly the reverse of that suggested by the original bivariate relationship—observed positives are really negatives and vice versa.

Given that one or more of these three potential changes (reduction, increase, change of sign) can occur when variables are either introduced or removed from a model, analysts need to be alert to these possibilities in their statistical practice. An important influence on what will happen is the extent of the interrelationships between included predictor variables. The degree of collinearity can readily be, though frequently is not, assessed by analysts using the Variance Inflation Factor (VIF) statistic (Allison 1999). The VIF for each independent variable can be obtained by regressing it against all others in the set being analysed, and then calculating (1/[1 − R^2^]). A VIF of 1.8 tells us that the variance of that predictor variable (i.e. its standard error) is 80% greater than would be the case with no collinearity effect: VIFs of 2.5 or greater are generally considered indicative of considerable collinearity suggesting that there will be difficulty in separating out the independent contribution of variables with such large VIFs—although some authors (e.g. Allison 1999, p. 142) put the cut-off much higher with a VIF at 10 or greater, a strategy not favoured in the context of the analyses reported here. (It should be stressed than this argument applies to situations where the independent variables are assumed to have parallel causal influences on the dependent, and that some do not come prior to others in a causal sequence, as is the case in analyses deploying the concept of mediating variables—for example, of age and social class being influences on attitudes, which in turn influence voting behaviour: the prior variables may have both a direct and an indirect, through the mediating variable, on the dependent: see Baron and Kenny (1986); Imai et al. (2010, 2011). In such cases, alternative analytical strategies—such as path analysis (Shipley 2009)—should be deployed). Alternatively, VanderWheele and Ding (2017) have suggested a procedure that estimates how strongly an unmeasured confounder would have to be related to both the dependent and the independent variable in order for that relationship to become insignificant/irrelevant—a somewhat different approach to the procedure suggested here which aims to identify those confounders.

Does it matter? In a discussion of ‘When can you safely ignore multicollinearity’ Allison (2012; see also O’Brien 2016) identified three situations when collinearity can be ignored:

- When the variables concerned are control variables in a regression model, whose coefficients are not to be interpreted, but the variables of interest do not display collinearity, either among themselves or with the control variables;
- One or more of the variables is a power of another variable included in the regression—for example, some regressions include both age and age^2^ as variables, and these are almost certain to be collinear; or
- The variables concerned are dummy variables representing variables with three or more categories.

But these do not apply in many cases. In much electoral analysis, even though control variables are included (age, social class, sex etc.) their coefficients are nevertheless often interpreted. Hence the need for care interpreting regression coefficients when collinearity may be present—and, as demonstrated here, even when that collinearity is relatively small there can be substantial impacts indicative of confounding although two independent variables are only relatively weakly correlated.

For clarity, the nature of the partial regression coefficients (the word ‘partial’ is almost invariably omitted in presentations) in any multiple regression equation needs to be fully appreciated. They indicate the relationship between the relevant independent variable and the dependent—holding constant the impact of all other independents. Thus, for example, if *Y* is being regressed against *X*_1_ and *X*_2_, then the partial regression coefficient between *Y* and *X*_1_ involves, in effect, the regression of the residuals of the regression of *Y* on *X*_2_ on the residuals of the regression of *X*_1_ on *X*_2_. What is frequently not recognised when such regression results are reported is that the greater the correlation between *X*_1_ and *X*_2_ the greater the likelihood that the relationship between *Y* and *X*_1_ is, in effect, modelling little more than random noise (i.e. in the residuals). The results may be—and often are—expressed as regressing *Y* on *X*_1_, holding constant the effect of *X*_2_, but if *X*_1_ and *X*_2_ are closely inter-related there is little left to analyse separately.

But how closely? The conventional wisdom—when it is applied—regarding collinearity in voting analyses suggests that it should only be addressed when the VIF values are relatively large; in other circumstances it is assumed, without any detailed investigation, that any relationships among two or more of the independent variables do not substantially influence the outcome of statistical modelling and hence the interpretation of the regression coefficients. Even where collinearity is neither ‘perfect’ nor ‘almost perfect’—as Baguley’s (2013) web tutorial describes it—correlations among the independent variables may create problems of confounding, as illustrated here. Care is thus needed in how model output is interpreted, and many results may require reconsideration of the model’s structure—as suggested by Kennedy (2008, pp. 196–202); a procedure—introduced several decades ago but rarely used, including in voting studies—is introduced that assists with such interpretation.

To illustrate those arguments, three examples are presented of analyses in which one or more of the impacts of confounding—spurious inflation; masking or suppression; reverse interpretation—occurs in standard analyses of voting behaviour. The first two—an ecological, ordinary least squares, analysis of voting for a political party in England and Wales,^2^ and a binary logistic regression analysis of party choice at an election to the Welsh National Assembly—illustrate that apparently relatively insubstantial inter-relationships among the independent variables can have a substantial confounding effect on a regression outcome. The final example uses a simulated data set, of a type widely used in some voting analyses, to illustrate how in some situations confounding can generate what can only be described as nonsense results. Throughout, the paper’s focus is on the one issue—confounding; it is assumed that the models are correctly specified and meet the other assumptions of the general linear model (such as an absence of autocorrelation in the residuals). Much attention is now paid to the use of diagnostics in the application of regression models (e.g. Fox 1991): this paper illustrates the importance of one such diagnostic tactic, whose application should remove a problem of mis-interpretation of regression outcomes.

## An ecological example: UKIP voting in England and Wales, 2015

The United Kingdom Independence Party’s (UKIP) success at recent British elections—notably for the European Parliament in 2014 and in the 2015 general election—is generally linked to the attractiveness of its right-wing populist appeal to those who have gained least from globalisation over recent decades in particular among: older people (especially males); those with few, if any, formal educational qualifications; and those living in areas with high levels of economic and social deprivation (see Goodwin and Milazzo 2015; Goodwin and Heath 2016; Clarke et al. 2017). To evaluate whether this was the case, the percentage of the votes cast for UKIP in each English and Welsh constituency at the 2015 general election was regressed against four variables derived from the 2011 census:^3^ the percentage of the adult population with either no or few (Level 1 only) educational qualifications;^4^ the percentage of the population aged 65 and over; the percentage of households with two or more deprivation characteristics;^5^ and the percentage of the adult population who were full-time students. (We are mindful of the need to avoid committing an ecological fallacy, because the relationships sought are between places not people.) The expectation was that each of the first three variables would be positively related to UKIP’s performance, whereas the fourth would be negatively linked. According to conventional analyses there is only a collinearity issue with two of those four variables, with VIF values of 3.8, 1.9, 3.7 and 1.7 respectively. The analyses reported here, however, show how the inter-relationships among all four have a confounding impact on the regression equation outcomes.

As a first stage, UKIP’s vote share was regressed against each of the four independent variables separately. Each was significantly related to the dependent variable, and with the expected sign (Table 1: Model 1 a–d): by far the strongest relationship—as shown by the r^2^ value—was with the qualifications variable. But did adding one of the other variables to a multiple regression also including qualifications substantially increase the model’s goodness of fit?

**Table 1:** Ordinary least squares regressions of the percentage voting UKIP by constituency in England and Wales at the 2015 UK general election

	a	b_1_Qual	b_2_Age	b_3_Deprive	b_4_Students	r^2^/R^2^
S*imple regressions using single independent variables*						
Model 1a	− 5.53	0.55				0.52
	(0.81)	(0.02)				
Model 1b	6.00		0.49			0.14
	(0.89)		(0.05)			
Model 1c	8.48			0.23		0.07
	(0.93)			(0.04)		
Model 1d	17.88				− 0.64	0.17
	(0.39)				(0.06)	
*Multiple regressions using pairs of independent variables (including Qual)*						
Model 2a	− 9.29	0.51	0.30			0.57
	(0.90)	(0.04)	(0.02)			
Model 2b	− 4.08	0.79		− 0.41		0.63
	(0.72)	(0.03)		(0.03)		
Model 2c	− 2.26	0.50			− 0.27	0.55
	(0.97)	(0.02)			(0.05)	
*Multiple regressions using three independent variables (including Qual)*						
Model 3a	− 7.40	0.50	0.26		− 0.11	0.57
	(1.27)	(0.02)	(0.04)		(0.05)	
Model 3b	− 4.37	0.78	0.02	− 0.40		0.63
	(0.97)	(0.3)	(0.05)	(0.04)		
Model 3c	− 5.48	0.85		− 0.48	0.14	0.63
	(0.91)	(0.04)		(0.04)	(0.06)	
*Multiple regression using all four independent variables*						
Model 4	− 6.14	0.84	0.04	− 0.46	0.14	0.63
	(1.19)	(0.40)	(0.05)	(0.05)	(0.06)	

	A	b_1_F_1_	b_2_F_2_			r^2^/R^2^
*Multiple regression using scores on the two factors identified in* Table 2 *as independent variables*						
Model 5	14.29	2.67	2.89			0.50
	(0.17)	(0.17)	(0.17)			

Figures in brackets are the standard errors of the regression coefficients

At the second stage (the second block of three equations in Table 1: Models 2a–c), therefore, each of the three other variables was added to a model that also included the qualifications variable—that with by far the highest correlation in the simple regressions. Compared to an r^2^ value of 0.52 when qualifications was the only independent variable included (Model 1a), the three R^2^ values all show an increase, by as much as 0.11 when household deprivation is the additional variable (in Model 2b). But two features of that last regression equation raise immediate concerns regarding confounding. First, the regression coefficient for qualifications increases from 0.52 (the first block in Table 1) to 0.79; and second—and very importantly—the (highly significant) coefficient for deprivation is positive (0.23) when that is the only variable regressed against UKIP performance but negative (− 0.41), and again highly significant statistically, when both variables are included. The correlation between the two independent variables is not especially large (r^2^ is 0.49 and the VIF involving those two variables alone is 1.96), but it is clearly sufficient to suggest that UKIP performed less well on average in the more deprived areas when qualifications are taken into account, whereas the regression with deprivation as the only independent variable indicated the opposite conclusion. Which is correct?

Of the other two-independent-variable regressions in that second block of Table 1, that including both qualifications and age (Model 2a) has a much smaller coefficient for the latter variable than in the single-variable model for age alone in the first block. That incorporating both student numbers and qualifications (Model 2c) also has a much smaller regression coefficient for the former variable than in the previous analysis (− 0.27 as against − 0.64 in Model 1d). (The separate r^2^ values between those two variables and that for qualifications were only 0.04 and 0.10 respectively; the VIFs were small—1.04 and 1.11 respectively—but the size of the regression coefficients changes substantially, although they are not statistically significant). Confounding with substantial changes in the estimated coefficient is thus common in these two-independent-variable regressions, despite the relatively weak collinearity.

At the third stage, two of the other three variables were added to that for qualifications in three three-independent-variable models (the third block in Table 1). The first of these—using qualifications, age and students (Model 3a)—suggests that the size of an area’s student population had much less impact on UKIP’s performance (albeit still negative) than the single-variable model (1d) for that variable showed. In the second—using qualifications, age and deprivation (Model 3b)—age is now statistically insignificant as an influence on UKIP’s vote share, and UKIP again apparently performed better the less deprived the area’s households. And when deprivation and students are the additional variables (Model 3c), the coefficient for each is the opposite of the expected.

When all four of the independent variables are included in a single equation (Model 4 in Table 1), three of the four are significantly related to UKIP’s performance, but two of them have an opposite sign to that expected. In many analyses, this would be the only model fitted—the four variables are ‘theoretically’ expected to be linked to UKIP’s vote share—and the interpretation would be that: UKIP’s performance increased the more adults in a constituency with no or minimal qualifications and the more students there were living there, but decreased the greater the proportion of households living in social-economic deprivation.

A change in the sign of the partial regression coefficient for an independent variable when a further independent variable is added to the regression model is not necessarily an indication of a problem. It may be the case that in areas with many old people students are more likely to vote for UKIP than is the case in areas with few old people, but if a model produces such a conclusion it should be explored further—as we illustrate here. We need to consider not just the change but also the potential reasons for that change.

### Refining the analysis

Is that interpretation a function not of the ‘true’ relationships between the four independent variables and UKIP’s vote share but rather a confounding consequence of the interrelationships among the four? If that is the case, one way forward—briefly identified by Kennedy (2008, pp. 197–198)—is to restructure the independent variables to remove the collinearity, using either principal components or factor analysis to replace the original variables by a new set of grouped, related variables. A principal components analysis of the four independent variables was thus undertaken, and the resulting two-component solution derived (varimax-rotated to obtain simple structure; i.e. each variable maximally-related to one of the two components). The resulting component loadings are shown in Table 2. With varimax rotation two clear pairs of interrelated variables emerge: qualifications and students on the first component (the more students in a constituency the fewer adults with no-or-minimal qualifications and vice versa); and age and deprivation on the second (the larger the percentage of old people in a constituency the more deprived households there are).

**Table 2:** Loadings on the principal components factor analyses of the four independent variables deployed in the regressions in Table 1

	Factor		Varimax rotated	
Variable/Factor	1	2	1	2
Qualifications	0.65	− 0.60	0.87	− 0.10
Age	0.82	0.53	0.34	0.91
Deprivation	0.31	0.92	− 0.30	0.93
Students	− 0.77	0.43	− 0.87	− 0.10

The component scores for each of those constructs for each constituency were calculated and used as the two independent variables in a fifth regression (Model 5 in Table 1). The highly significant regression coefficients show—as expected—that UKIP performed better in constituencies with more old people and deprived households (i.e. the second component), and also in those with more adults with no-or-minimal qualifications; it performed less well, the more students there were living in a constituency. The hypothesised patterns emerged—but they didn’t in the type of modelling normally deployed, where all four variables are entered in a single regression.

What is the relative strength of the four independent variables as influences on the dependent, taking the interrelationships into account? Kennedy (2008) does not address this issue, instead focusing on the interpretability of the components. However, the relative strength of the individual variables can be assessed using a procedure introduced separately by Riddell (1970) and by Sanint (1982; see also Massy 1965), but little used since (see Johnston et al. 2004),^6^ in which a reconstituted standardised regression coefficient for each independent variable can be derived by summing the product of its loading on each component and the regression coefficient for that component across all components (in this case two). The resulting standardised coefficients are:

**Table:** 

Qualifications	2.03	Age	3.53
Deprivation	1.89	Students	− 2.61

In relative terms, therefore, UKIP’s vote share increased most as the percentage of the constituency population aged 65 and over increased, then as the percentage of students decreased, then as the percentage of adults with no-or-minimal qualifications increased, and finally as the percentage of deprived households increased: the expected patterns with the relative importance of each isolated. (This conclusion may appear partly counter-intuitive, given the much higher correlation between UKIP’s vote share and the qualifications variable than with the age variable in the first block of Table 1. But there is more variation in the latter variable across the constituencies; the coefficient of variability [the standard deviation as a percentage of the mean] is 25% for the age variable, but only 20% for qualifications).

An alternative strategy might be to deploy all four independent variables but enter them in a stepwise model. If this is done, however, at the first stage the qualifications variable enters; at the second, deprivation is added, but with an unexpected negative regression coefficient; and the other two variables would be excluded as insignificantly related to the dependent. A further alternative might be to include all four variables in the one model but also to add the three interactions involving each of the other three with qualifications. Only one of the three interaction relationships is statistically significant—the more old people and poorly-qualified people in a constituency, the better UKIP’s performance. The message is that multiple models are needed to appreciate what is going on, stepwise modelling is not an automatic solution, and combining variables in meaningful ways can be helpful in teasing out the underlying relations.

Usual practice in the statistical analysis of voting patterns would involve selection of the four independent variables and fitting a regression model incorporating all four—with the result shown in the fourth block of Table 1. This would then be interpreted as indicating not only that, as anticipated, UKIP support increased as the percentage of poorly-qualified individuals in a constituency increased but also that: UKIP support unexpectedly decreased the more deprived households there were in a constituency; UKIP support unexpectedly increased the more students there were in a constituency; and there was no significant relationship between UKIP support and the percentage of a constituency’s population who were old—three of those findings being contrary to expectations. This is because of the confounding impact of inter-relationships among those four independent variables, even though the VIF values do not suggest major issues relating to collinearity; low VIF values are not indicators of the absence of confounding effects. If the procedure introduced here involving the use of principal components analysis to take those inter-relationships into account is deployed, however, then the four hypotheses underpinning the selection of independent variables are confirmed—which is almost certainly the ‘true’ situation as against that reached using standard practices set out in Table 1.

## Analysing survey data using logistic regression: voting for the National Assembly of Wales, 2011

Many electoral studies are based on survey data exploring, for example, the determinants of party choice through either binomial or multinomial logistic regressions. To illustrate the impact of independent variable interrelationships in such investigations, we use data from the 2011 Welsh Electoral Study (with 1963 respondents^7^) to explore determinants of voting for Labour, the country’s largest party and the dominant member of a Welsh National Assembly coalition government (with Plaid Cymru) in the years preceding the election. The dependent variable—Y—is thus a binary coded 1 if the respondent voted Labour and 0 otherwise in the 2011 constituency contests.

In many such studies, instead of including a substantial number of socio-economic and/or demographic variables as potential influences on party choice a variable such as either how the respondent voted at the last election or party identification is included to assimilate all such influences (i.e. as a composite control variable). Further variables then look at the proximate influences on the vote—what led some who voted for the party last time to desert it at the subsequent election, for example, or, for some of those who voted otherwise at the first election of the pair, what stimulated them to switch their allegiance at the next contest. Thus, the first independent variable in this analysis—X_1_—is coded 1 if the respondent voted Labour at the Welsh Assembly election in 2007 and 0 otherwise, so we are modelling change. The result—the odds ratio in the first regression in Table 3 (Model 1a)—shows the expected strong positive relationship; those who voted Labour in 2007 rather than vote in any other way then were 21.858 times more likely to vote Labour in 2011 than in any other way (i.e. for either another party or to abstain).

**Table 3:** Logistic regressions of voting labour at the 2011 constituency-level elections to the National Assembly of Wales

	a	X_1_	X_2_	X_3_
*Model 1a*				
Coefficient	− 0.089	3.085		
SE	(0.065)	(0.129)		
Exponent	0.915	21.858		
r^2^		0.437		
*Model 1b*				
Coefficient	− 0.350		1.754	
SE	(0.053)		(0.106)	
Exponent	0.705		5.780	
r^2^			0.191	
*Model 1c*				
Coefficient	− 0.356			1.149
SE	(0.054)			(0.107)
Exponent	0.701			3.154
r^2^				0.079
*Model 2a*				
Coefficient	− 0.217		1.574	0.703
SE	(0.059)		(0.110)	(0.118)
Exponent	0.805		4.824	2.020
R^2^				0.212
*Model 2b*				
Coefficient	0.098	2.889	1.399	
SE	(0.069)	(0.134)	(0.129)	
Exponent	1.998	17.974	4.051	
R^2^			0.492	
*Model 2c*				
Coefficient	0.081	2.990		0.809
SE	(0.072)	(0.130)		(0.135)
Exponent	1.084	19.882		2.246
R^2^				0.454
*Model 3*				
Coefficient	0.173	2.848	1.285	0.444
SE	(0.074)	(0.134)	(0.134)	(0.143)
Exponent	1.189	17.262	3.616	1.559
R^2^				0.496

The independent variables are *X*_1_ voted Labour in 2007, *X*_2_ strongly likes the labour party *X*_3_ strongly likes the Labour party leader, Carwyn Jones

Other variables commonly included in such analyses ask respondents how well they like either or both of the party itself and its leader in the legislature—with the latter often presented as a short-cut heuristic deployed by voters (Clarke et al. 2010). The X_2_ and X_3_ in these analyses are coded, respectively, 1 if the respondent strongly liked the party (a score of 8 or greater on an 11-point scale from 0–10) and 1 if the respondent strongly liked the party’s leader (Carwyn Jones)—and 0 otherwise. The results of the two regressions deploying those variables separately (Model 1b, c) again show the expected positive relationships—much stronger for liking the party than for liking its leader (Table 3). The two are not strongly interrelated, with a VIF (using the Nagelkerke r^2^ value) of only 1.27: nevertheless, the partial regression coefficient for X_3_ in Model 2a is substantially smaller at 0.703 than the 1.149 recorded in the regression of X_3_ alone on Y—which would be interpreted as saying that the impact of the Labour party leader’s image on whether respondents voted Labour in 2011 was substantially reduced once the impact of his party’s image was taken into account.

Those variables could be related to the control variable, however, with whether respondents voted Labour in 2007; previous Labour voters are more likely than those who did not vote Labour at the previous election to like both the party and its leader subsequent to the election (an endogeneity point discussed in detail by Evans and Chzhen 2016). The VIFs for the three variables are only 1.19, 1.28 and 1.20 respectively; nevertheless, the two regressions including either X_2_ or X_3_ along with X_1_ in Table 3 bear out this expectation. The coefficients and exponents for both X_2_ and X_3_ are substantially smaller in their respective two-independent-variable multiple regressions than in the simple regressions of either X_2_ or X_3_ with Y; holding previous vote constant, party and leader images have substantially smaller influence on voter choice at the next election than when that is not taken into account. Finally, when all three variables are included—Model 3 in Table 3—those coefficients are further reduced, especially that for X_3_; it remains significantly linked to Y, but with an exponent only half of its size when X_3_ is regressed against Y alone.

Apart from general evaluations of parties and their leaders, many studies also ask respondents to assess the governing party’s (or parties’) performance on particular issues. The 2011 Welsh Election Study included six such assessments, of running Wales generally, and of handling the major issues of the previous 4 years—the NHS, schools, University tuition fees, the economy, and Welsh interests. These are introduced as binary variables X_4_–X_9_, each coded 1 for a good performance and 0 otherwise. There are interrelationships among these variables—not surprisingly those who rated the government’s performance positively on one policy issue were more likely to do so on the others—but only one of the VIF values exceeds the ‘standard’ 2.50 threshold (for X_4_–X_9_ respectively they are 1.83, 2.58, 2.30, 1.91, 2.42 and 2.45)

The first six binary logistic regressions in Table 4 (Models 1a–f) show that individually all six variables were both positively and statistically significantly related to voting Labour in 2011, with five of the exponents averaging c.3.57 and the other (for running Wales well) twice that size. But interrelationships clearly have an impact, as shown by the next two regressions (Models 2a–b). The first includes all of the policy-specific areas—X_5_–X_9_; all of their regression coefficients are substantially smaller than those in their single-variable regressions above and two of them, for the school and economy policy areas (X_6_ and X_8_), are statistically insignificant. When the general variable X_4_ is added (Model 2b), not only do the coefficients for X_5_–X_9_ reduce further, with four of them statistically insignificant, but in one case—variable X_8_, handling of the economy—the insignificant coefficient is also negative.

**Table 4:** Further logistic regressions of voting labour at the 2011 constituency-level elections to the National Assembly of Wales

	a	X_4_	X_5_	X_6_	X_7_	X_8_	X_9_
*Model 1a*							
Coefficient	− 0.471	2.035					
SE	(0.053)	(0.106)					
Exponent	0.625	7.649					
r^2^		0.258					
*Model 1b*							
Coefficient	− 0.538		1.371				
SE	(0.050)		(0.100)				
Exponent	0.584		3.941				
r^2^			0.132				
*Model 1c*							
Coefficient	− 0.361			1.220			
SE	(0.053)			(0.106)			
Exponent	0.697			3.387			
r^2^				0.092			
*Model 1d*							
Coefficient	− 0.630				1.326		
SE	(0.050)				(0.100)		
Exponent	0.532				3.768		
r^2^					0.125		
*Model 1e*							
Coefficient	− 0.372					1.189	
SE	(0.053)					(0.105)	
Exponent	0.698					3.284	
r^2^						0.088	
*Model 1f*							
Coefficient	− 0.599						1.244
SE	(0.049)						(0.099)
Exponent	0.549						3.470
r^2^							0.111
*Model 2a*							
Coefficient	− 0.499		0.633	0.224	0.671	0.191	0.353
*Model 3b*							
SE	(0.060)		(0.135)	(0.141)	(0.124)	(0.141)	(0.136)
Exponent	0.607		1.884	1.251	1.955	1.211	1.423
R^2^							0.179
*Model 2b*							
Coefficient	− 0.497	1.663	0.258	0.088	0.538	− 0.140	0.111
SE	(0.063)	(0.129)	(0.148)	(0.150)	(0.132)	(0.153)	(0.146)
Exponent	0.609	5.273	1.294	1.092	1.712	0.869	1.117
R^2^							0.280

	a	X_1_	X_2_	X_3_
*Model 3*				
Coefficient	0.031	2.597	1.056	0.045
SE	(0.086)	(0.139)	(0.138)	(0.151)
Exponent	1.031	13.424	2.876	1.046

	X_4_	X_5_	X_6_	X_7_	X_8_	X_9_
Coefficient	0.940	0.159	0.070	0.558	− 0.209	0.113
SE	(0.160)	(0.181)	(0.179)	(0.160)	(0.184)	(0.176)
Exponent	2.561	1.172	1.073	1.747	0.812	1.120
R^2^						0.540

The independent variables: are *X*_1_ voted Labour in 2007, *X*_2_ strongly likes the Labour party, *X*_3_ strongly likes the Labour party leader, Carwyn Jones, *X*_4_ Labour at good running Wales, *X*_5_ Welsh government handled NHS well, *X*_6_ Welsh government handled schools well, *X*_7_ Welsh government handled University tuition fees well, *X*_8_ Welsh government handled economy well, *X*_9_ Welsh government handled Welsh interests well

Finally, a full regression including all nine independent variables (Table 4, Model 3) further exemplifies the confounding impact of interrelationships on the interpretation of the links between the independent variables and voting for the Labour party’s candidates. Five of the nine regression coefficients are statistically insignificantly larger or smaller than zero, including that for the respondents’ evaluations of the party’s leader (and one of them is again negative). Additionally, all of the exponents in that multiple regression are substantially smaller than that for the particular variable in the relevant simple regressions—by more than one-half in all cases except that for variable X_1_, whether the respondent voted for a Labour candidate in 2007.

There are clearly sufficiently strong interrelationships in this data set, despite the low VIF values, to influence the regression outcomes and thus the interpretation of how the various factors influenced voter choice at the 2011 National Assembly of Wales election; there is substantial confounding. This is further illustrated by again deploying principal components factor analyses. Three were undertaken (Table 5): the first two (for variables X_1_–X_3_ and X_4_–X_9_ respectively) each resulted in single-component solutions, accounting for 53 and 60% of the variation respectively. For the first, all three variables had a loading of 0.70 or greater on that component, and the scores related to it (FI_a_) were positively related to whether respondents voted Labour in 2011 (the first regression reported in Table 5). All six variables had loadings of 0.73 or greater in the second analysis, and the scores (FI_b_) were also positively related to Y. When both sets of scores were included in a regression, each was positively and significantly related to the probability of a Labour vote in 2011, with variation on FI_a_ having more than twice the impact than variation on FI_b_.

**Table 5:** Loadings from the principal components factor analyses of the data analysed in Table 4, and the results of logistic regression analyses using the related factor scores as independent variables to predict voting labour at the 2011 constituency-level election to the National Assembly of Wales

Variable	FI_a_	FI_b_	FI_ab_	FII_ab_
X_1_	0.702		0.231	0.787
X_2_	0.775		0.315	0.673
X_3_	0.703		0.332	0.683
X_4_		0.734	0.690	0.617
X_5_		0.819	0.815	0.381
X_6_		0.771	0.787	0.250
X_7_		0.737	0.734	0.352
X_8_		0.784	0.798	0.260
X_9_		0.803	0.805	0.341

Y = − 0.763 + 1.429FI_a_ r^2^ = 0.403

(0.058) (0.066)

Y = − 0.707 + 0.892FI_b_ r^2^ = 0.211

(0.053) (0.052)

Y = − 0.820 + 1.244FI_a_ + 0.518FI_b_ R^2^ = 0.438

(0.061) (0.068) (0.061)

Y = − 0.833 + 0.464FI_ab_ + 1.328FII_ab_ R^2^ = 0.449

(0.062) (0.060) (0.069)

When all nine variables were included in a single principal components analysis, two components, together accounting for 59% of the variation, were extracted and simple structure was obtained using a direct oblimin rotation. The first component has its heaviest loadings for variables X_4_–X_9_, and the second for X_1_–X_3_ although there were some substantial cross-loadings on both components—notably for X_4_. Regressing the two sets of component scores on Y (the final regression in Table 5), shows both to have a positive impact; variation in Labour voting was greater relative to (standardised) variation in previous vote and party/leader image (FII_ab_) than it was to variations in evaluations of government performance (FI_ab_).

As was concluded from the ecological regression example, therefore, substantial confounding effects—some involving spurious inflation, some masking or suppression, and some reverse interpretation—appear in this set of multinomial regression analyses, despite the low levels of collinearity among the variables. Care is needed when running such analyses, therefore: confounding can mask the true relationships unless it is taken into account in the model structure.

## A further logistic regression example: towards nonsense results

To exemplify further confounding and its impact on the nature of regression outcomes—some difficult to interpret, others nonsensical—we use a simulated data set comprising 1500 observations.^8^ (This comprises 20 separate observations—shown in the “Appendix” table—repeated 75 times.)

The dependent variable in this data set—*Y*—is voting for Labour (coded 1 if voted Labour and 0 otherwise). There are two independent variables: *X*_1_—whether the respondent is a member of the Working Class (coded 1 if Working Class and 0 otherwise); and *X*_2_—whether the respondent considers Labour is the best party to tackle the problems of the economy (coded 1 if Labour is best and 0 otherwise). The latter of those independent variables appears in four different scenarios—*X*_21_, *…,* *X*_24_—each of which has a closer correlation with *X*_1_ than the previous version. (The distribution of those considering Labour best placed to handle the problems of the economy across the 1500 respondents to the hypothetical survey has been varied to alter the correlation of that variable with *X*_1_.) The correlations (Nagelkerke r^2^) between *X*_1_ and each of those four, derived from binary logistic regressions, are:

$$X_{1,} X_{21} \;0.020;\quad X_{1,} X_{22} \;0.149;\quad X_{1,} X_{23} \;0.375;\quad X_{1,} X_{24} \;0.662$$

There is virtually no correlation between the two variables in the first example, therefore, and only a slight one in the second; correlation is more substantial in the third example, and even more so in the fourth. The VIF values are thus:

$$X_{1,} X_{21} \;1.02;\quad X_{1,} X_{22} \;1.18;\quad X_{1,} X_{23} \;1.60;\quad X_{1,} X_{24} \;2.95$$

which suggest that collinearity and confounding should only be a problem when *Y* is regressed against *X*_1_ and *X*_24_.

The first binomial regression in Table 6 (Model 1) shows a positive, significant relationship between class membership and voting Labour, and for the next four regressions (*Economic Competence and Vote*: Models 2a–d) each shows a similar relationship (though with varying intensity) between opinions on Labour’s ability to manage the economy and voting Labour; all of those relationships are positive and statistically significant (i.e. the regression coefficient is at least twice the size of its standard error).

**Table 6:** Logistic regressions of the data in “Appendix”

	a	X_1_	X_21_	X_22_	X_23_	X_24_
*Class and Vote*						
Model 1						
Coefficient	− 0.458	1.492				
SE	(0.058)	(0.116)				
Exponent	0.632	4.444				
Nagelkerke r^2^		0.149				.
*Economic Competence and Vote*						
Model 2a						
Coefficient	− 0.549		0.523			
SE	(0.056)		(0.112)			
Exponent	0.577		1.687			
Nagelkerke r^2^			0.020			
Model 2b						
Coefficient	− 0.347			4.277		
SE	(0.087)			(0.173)		
Exponent	0.707			72.000		
Nagelkerke r^2^				0.662		
Model 2c						
Coefficient	− 0.347				4.277	
SE	(0.087)				(0.173)	
Exponent	0.707				72.000	
Nagelkerke r^2^					0.662	
Model 2d						
Coefficient	− 0.394					2.621
SE	(0.066)					(0.131)
Exponent	0.674					13.750
Nagelkerke r^2^						0.375
*Class, Economic Competence and Vote*						
Model 3a						
Coefficient	− 0.409	1.456	0.392			
SE	(0.060)	(0.117)	(0.119)			
Exponent	0.664	4.288	1.480			
Nagelkerke R^2^			0.158			
Model 3b						
Coefficient	− 0.261	0.846		4.110		
SE	(0.089)	(0.179)		(0.175)		
Exponent	0.771	2.330		60.957		
Nagelkerke R^2^				0.672		
Model 3c						
Coefficient	− 0.458	− 19.433			23.122	
SE	(0.089)	(2289.293)			(2289.293)	
Exponent	(0.632)	0.000			0.000	
Nagelkerke R^2^					0.700	
Model 3d						
Coefficient	− 0.458	− 20.121				22.423
SE	(0.067)	(3229.065)				(3229.065)
Exponent	0.632	0.000				5,474,103,965.0
Nagelkerke R^2^						0.428

The final block of four regressions in Table 6 (Models 3a–d) reports multiple regressions between voting Labour and whether the respondent is a member of the Working Class plus one of the four versions of *X*_2_, which are increasingly correlated with *X*_1_, as shown above. In the first case—*X*_21_, with virtual nil correlation between the two (0.020)—the two independent variables clearly make additive contributions to a statistical explanation of variation in the values of *Y*; the coefficient, standard error and exponent for *X*_1_ are virtually unchanged from those in the first regression in Table 6, and the R^2^ value is (slightly) increased.

The next regression—Model 3b—replaces *X*_21_ by *X*_22_, which has a higher correlation with *X*_1_—though not large (0.149: VIF 1.18). Nevertheless, the coefficient for *X*_1_ is reduced by about 40% compared to the regression with *X*_21_ and the associated exponent is almost halved; there is an extremely large coefficient, and associated exponent, for *X*_22_.

The final two regressions (Models 3c–d), involving *X*_23_ and *X*_24_, produce results that can only be considered nonsensical, although the VIFs suggest that problems should only appear for that with *X*_24_. There are very large (though statistically insignificant) coefficients for *X*_1_ and comparable large (again statistically insignificant) coefficients (with meaninglessly large exponents) for *X*_23_ and *X*_24_. The correlations between *X*_1_ and each of the other two variables mean that only residual noise is being regressed against *X*_1_ once the common variance shared by the two collinear variables is held constant—and the result is very substantial spurious inflation, producing nonsense results.

## Conclusions

Many ecological- and individual-level analyses of voting behaviour use multiple regressions with a considerable number of independent variables but few discussions of their results pay any attention to the impact of collinearity among those independent variables, let alone report VIF values. Very few indeed explore various combinations of independent variables in their data to reveal the impact of collinearity and identify the likely impact of any one independent variable on the dependent; whether the relationships between the individual independent variables and the dependent are affected by confounding, and therefore difficult to interpret, is very rarely addressed. More importantly, as the examples in this paper have illustrated, even where collinearity is low substantial confounding can nevertheless occur as a result of interrelationships among the variables included in a model. Because most analysts only report the final model (and may have done no prior explorations of those interrelationships of the type reported here) it is rarely clear whether the results incorporate any substantial confounding that substantially impacts upon interpretations of the size and sign of partial regression coefficients and their statistical significance—and hence on the substantive and theoretical appreciation of the empirical tests.

Three examples have been used here to indicate the potential pitfalls of such practice. With little or only mild collinearity the impact on the interpretation should be slight, according to ‘standard practice’; regression coefficients may change in their size reflecting the results of partialling out the effect of other variables, but the standard errors are not inflated and considerable confidence can be expressed in the interpretations; there is little or no confounding. But as the links between independent variables strengthen (even though statistical tests suggest minimal collinearity) unexpected results appear: change in the direction of the regression coefficients, for example; increase in the standard errors; and, in logistic regressions, inflation in the values of the exponents associated with the regression coefficients, in some cases to nonsense levels.

All of this suggests care is needed in conducting such analyses. One regression model incorporating all of the selected independent variables should not be run and then reported without careful exploration, involving not only calculation of the VIF values but also running regressions with only some of the variables included. This could lead to decisions to eliminate some of the independent variables from the final version (one of Kennedy’s—2008—‘What to do’ suggestions) but if it is considered necessary to include them all to assess their joint impact an approach such as that deployed here using principal components analysis might be used. This approach, rarely used in the behavioural social sciences, offers a clear way forward in the analysis of voting patterns that avoids any confounding impacts of inter-relationships among the independent variables and provides a much clearer test of the strength of the impact of each independent variable on the dependent (as in Johnston et al. 2017)—rather than the analysis of residual noise that can characterise partial regression equations.

This paper has delivered a clear warning to electoral analysts (and other social scientists conducting observational research) using quantitative methods, notably regression. Confounding can have a substantial impact on the nature of model results and how they are interpreted in the light of prior expectations; indeed, confounded relationships could be the norm and interpretations open to doubt.^9^ Exploration of data by running several separate regressions with different variable combinations might be informative and make conclusions more insightful. Just because a coefficient is negative might not indicate the ‘true’ relationship between one variable and another—ceteris paribus!

While we have concentrated on the scale of the changes that come about as variables are either introduced to or removed from a model, it is also important to see this in a wider context. There is a large literature (e.g. Baron and Kenny 1986; MacKinnon 2008; Hayes 2013; VanderWeele 2015) that distinguishes between the *conceptual* status of the introduced variable in terms of confounders, mediators and modifiers. Changes involving one or more of reduction, increase, and reversal of sign in the original relation do not necessarily mean that the introduced variable is a confounder. Confounders are a nuisance and need to be nullified to prevent distortion of results; their impact occurs when the third variable is associated with both the exposure—the main predictor of interest—and the outcome but conceptually does not lie on the ‘causal’ pathway from the exposure to the outcome. Mediator variables, like confounders, show associations with both the exposure and the outcome, but are seen as lying on the causal pathway between exposure and outcome—a mediator variable is one that explains the relationship between the two other variables. Mediators are seen as intervening variables that produce the outcome; changes in the exposure lead to changes in the mediator which in turn result in changes in the outcome. The general statistical procedure for evaluating mediation is that the relation between exposure and outcome should be reduced after including the mediator variable. This reduction of original association when the third variable is included is not spurious, rather we have the development of an explanation. Finally, with effect modification, the third variable is interacting with exposure to modify the effect so that an exposure has a different effect among different subgroups. Effect modification is associated with the outcome but not the exposure. The results are not spurious but of real interest as the nature of the effect differs according to the presence of a third factor. In practice, in statistical analysis moderators are simply interaction terms that change the nature of the effect of the exposure on outcome.

The importance of this concluding discussion is that it is not just a technical matter of including variables and their interactions in a multiple regression-like model when exploring the multivariate relationships between variables but the nature of the ‘web of causation’ and the conceptual status of variables in the modelling should be carefully considered. To take two examples: including behavioural variables between class and voting and the apparent disappearance of the class effect should not be seen as confounding but rather that both class and behaviours are causally related, with the latter mediating the underlying relationship. Much analysis has been concerned with just the main effects when interactions are key to understanding: gender may make little difference but gender in interaction with age may (Jones et al. 2016). In sum, models should be carefully conceived and when they are fitted the results of the empirical analyses should be rigorously assessed to ensure that the ‘true’ patterns are appreciated: simply either fitting models with all of the model variables included or only modifying them when VIF values indicate substantial collinearity is not sufficient—validity is an argument not a statistic.

The message from this cautionary tale, therefore, is that in multiple regression analyses—as illustrated here with studies of voting behaviour—exploratory procedures should be deployed when empirically testing models in which the outcome is believed to be influenced by a number of contributory factors (independent variables) that are not structured in a causal path. Those procedures should:

- Check whether there is substantial collinearity among the independent variables;
- Explore whether there are confounding effects created by the inter-relationships among the independent variables that either apparently spuriously inflate or mask/suppress (even alter the direction of the relationship with) the apparent influence of one of more of the variables by conducting separate regressions using subsets of the independent variables only; and if those explorations indicate considerable confounding effects
- Adopt an alternative analytical procedure, such as that introduced here using principal components factor analysis, to circumvent those confounding effects and thereby identify the ‘true’ relationships.

Following these steps is in line with the general strategy set out by Franzosi (1994, p. 21) of preliminary analysis (getting to know the data), followed by confirmatory analysis (model testing) and then interior analysis (model checking). In particular, his final step provides ‘the necessary assurances about the basic soundness of the model’; the examples presented here have illustrated the importance of doing this and avoiding mis-interpretations of model outcomes.

## Appendix

See Table 7.

**Table 7:** The constructed data set

Observation	*X1*	*Y*	*X*_21_	*X*_22_	*X*_23_	*X*_24_
1	1	1	0	1	1	1
2	1	1	1	1	1	1
3	1	1	0	1	1	1
4	1	1	0	1	1	1
5	1	0	1	0	1	1
6	1	0	0	0	0	1
7	1	0	1	0	0	0
8	0	1	0	1	1	1
9	0	1	1	1	1	0
10	0	1	1	0	0	0
11	0	0	1	1	0	0
12	0	0	1	0	0	0
13	0	0	0	0	0	0
14	0	0	0	0	0	0
15	0	0	0	0	0	0
16	0	0	0	0	0	0
17	0	0	0	0	0	0
18	0	0	0	0	0	0
19	0	0	0	0	0	0
20	0	0	0	0	0	0

## Footnotes

1 Neither collinearity nor confounding was included in King’s (1986) discussion of the more common conceptual statistical mistakes in quantitative political science research.

2 Despite the increasing availability of survey data ecological analyses remain popular for the insights they provide into patterns of voting behaviour.

3 Scotland is omitted because UKIP was not strong and did not campaign intensively there, at an election that focused almost entirely on the Scottish National Party and its claims for both independence and retained membership of the European Union (which latter UKIP strongly opposed).

4 Educational qualifications in the UK are placed on a nine-point scale by the UK government: those of level-5 and above (i.e. degree and above) are combined here. See https://www.gov.uk/what-different-qualification-levels-mean/list-of-qualification-levels—accessed 18 July 2017.

5 The Office of National Statistics takes four measures of household deprivation—on employment, education, health and disability, and household overcrowding—and groups households as to the number of those four on which they are classified as deprived (for more information see Office for National Statistics 2014).

6 Sanint’s paper has only been cited on six occasions according to Google Scholar; Riddell’s has been cited 40 times, but few of those citations relate to the methodological issue; Massy’s paper has 738 citations, however. The paper by Johnston et al. (2004) has 51 citations, but again very few refer to the methodological issue addressed here. Apart from that latter paper, no example has been identified where the Riddell/Sanint procedure has been applied in studies of voting behaviour, either ecological or individual. (The citation data have been obtained from Google Scholar—http://scholar.google.co.uk/—accessed 19 July 2017).

7 We are grateful to Roger Scully for making these data available to us.

8 Although the problem illustrated here has been identified in ‘real’ data sets, its nature is more readily appreciated through a simulated set.

9 As one web dictionary argues—in the context of drug trials: http://stattrek.com/statistics/dictionary.aspx?definition = confounding (accessed 15 February 2017)—‘Confounding occurs when the experimental controls do not allow the experimenter to reasonably eliminate plausible alternative explanations for an observed relationship between independent and dependent variables’.
